# Coronavirus Disease (COVID-19–SARS-CoV-2) and Nutrition: Is Infection in Italy Suggesting a Connection?

**DOI:** 10.3389/fimmu.2020.00944

**Published:** 2020-05-07

**Authors:** Hellas Cena, Marcello Chieppa

**Affiliations:** ^1^Department of Public Health, Experimental and Forensic Medicine-Dietetics and Clinical Nutrition Laboratory, University of Pavia, Pavia, Italy; ^2^Clinical Nutrition and Dietetics Service, Unit of Internal Medicine and Endocrinology, ICS Maugeri IRCCS, Pavia, Italy; ^3^National Institute of Gastroenterology “S. de Bellis”, Institute of Research, Castellana Grotte, Italy; ^4^Depatrment of Immunology and Cell Biology, European Biomedical Research Institute of Salerno EBRIS, Salerno, Italy

**Keywords:** COVID-19, nutrition, polyphenols, diabetes, incidence, cytokine storm

## Abstract

Novel coronavirus disease (COVID-19) was declared a global pandemic on March 11, 2020. The outbreak first occurred in Wuhan, Hubei, China, in December 2019 and hit Italy heavily in February 2020. Several countries are adopting complete or partial lockdown to contain the growth of COVID-19 infection. These measures may affect people's mental health and well-being but are necessary to avoid spreading the pandemic. There has been a gradual increase in studies exploring prevention and control measures, and we recommend paying close attention to nutrition, which may contribute to modulating some important consequences of COVID-19 infection, as such pro-inflammatory cytokine storm.

## Introduction

Novel coronavirus disease (COVID-19) spread to all regions of Italy on January 31, 2020. The northern region of Lombardy was identified as the center of the two main Italian clusters of cases. On March 11, 2020, the Director-General of the WHO declared COVID-19 a global pandemic as the virus spread rapidly from China to the rest of the world, particularly Europe (1). Currently, the scientific community is sharing potentially useful data to treat patients and protect the population, but the details of COVID-19 infection are still largely unknown, and thus options for risk assessment and pharmacological intervention have only been partially developed.

By March 26, more than 511,603 confirmed cases of COVID-19 had been reported, of which 80,589 were registered in Italy, so far the country with the highest mortality rate (8.215) (2). Four Regions in Italy have reported 59,173 cases of COVID-19, including 34,889 confirmed infections solely in Italy's epicentral Lombardy region, corresponding to more than a third of the total number of infected people in the entire country (Ministry of Health). Italy's mortality rate may be partially explained by the country's relatively higher proportion of older people.

Similarly to what has been observed in China, the most common symptoms were fever, cough, and fatigue (3). Acute respiratory distress syndrome (ARDS) was the main cause of death (4). Older adults and people with chronic illness are most vulnerable to the worst effects of the disease.

Knowledge about novel COVID-19 is based on a few months of observation and some similarities to Severe Acute Respiratory Syndrome (SARS) and to Middle East Respiratory Syndrome coronavirus (MERS-CoV). Despite the lower case fatality rate than MERS and SARS, COVID-19 has so far proven extremely contagious. Moreover, a significant percentage of those who are infected require hospitalization in an intensive care unit.

Here we speculate on a possible link between nutritional status and COVID-19 mortality, based on data emerging from the Italian National Health System. Furthermore, while waiting for clinical trials to shed light on the clinical efficacy and beneficial effects of antibodies and anti-inflammatory cytokines, we highlight nutritionally derived products that may inhibit the inflammatory cytokine secretion caused by COVID-19 infection.

## Diabetes And COVID-19 Infection

Subjects with diabetes are at risk of infections and have severe disease when infected with a respiratory virus, showing an increased risk of mortality (5).

Data from MERS-CoV, which broke out in 2012 in Saudi Arabia, showed that the severity and length of the pulmonary pathology observed were increased in those affected by type 2 diabetes, which likely deregulates immune response (6). Data on COVID-19 in patients with diabetes is limited at present (5). The Chinese Centre for Disease Control and Prevention published a report of 72,314 cases of COVID-19, showing an increased mortality rate in subjects with diabetes (7).

Diabetes is the most common comorbidity observed in COVID-19-positive deceased patients in Italy after hypertension (8). Available data on pre-existing comorbidities updated on April 9, 2020, and extracted from clinical charts showed hypertension and diabetes before hospitalization in 69.9% (*n* = 1,015) and 33.8% (*n* = 462) of COVID-19-positive deceased patients, respectively (9). The same data showed that among 18,366 COVID-19-positive deceased patients, 61.0% (*n* = 886) presented three or more comorbidities that had been diagnosed before COVID-19 infection, 20.7% (*n* = 301) had two, 18.8% (*n* = 215) had one, and only 3.5% (*n* = 51) had no pre-existing pathology (9).

Diabetes is prevalent in our population, and most patients with diabetes type II are overweight or affected by obesity (10). However, obesity is not included within the WHO “five-by-five” framework of non-communicable diseases (NCDs) and risk factors, and data on BMI are not collected in a standardized manner^1^. Unfortunately, we do not yet have weight, height, and waist circumference data for all patients with laboratory-confirmed COVID-19, and, therefore, we cannot disentangle the effects of adiposity on lung function and immune response to viral infection. Excess body weight and increased visceral adiposity are habitually associated with metabolic alterations such as insulin dysregulation, high fasting glucose levels, hyperlipidemia, or systemic hypertension, which cause dysregulation of the immune system through mediation in various immune, metabolic, and thrombogenic responses. However, the clinical impact of this immune dysregulation on susceptibility to and severity and outcome of viral infections and on lung function is not yet clearly understood (11, 12).

Nevertheless, preliminary data from GiViTi (https://giviti.marionegri.it/covid-19/) presented on March 31, 2020, showed a high prevalence of obesity (26%) and overweight (41%) in 928 Italian patients, median age 65 years, from 76 different Italian ICUs, confirming evidence available so far in the literature. Recent data on patients with laboratory-confirmed COVID-19 treated at an academic health institution in New York City, the epicenter of the COVID-19 outbreak in the United States, between March 1, 2020, and April 2, 2020, with follow up through April 7, 2020 (13, 14) showed that obesity, after age, was linked to more severe coronavirus cases, with a substantially higher odds ratio than any cardiovascular or pulmonary disease.

Obese and obese-diabetic subjects undergo modifications of the innate and adaptive immune response at different phases, characterized by a state of chronic, and low-grade inflammation and a high basal concentration of several pro-inflammatory cytokines such as alpha-TNF, MCP-1, and IL-6, leading to a defect in innate immunity (14). Recent evidence indicates that obesity not only increases the risk of infection and of complications for the individual but also increases the chance of appearance of a more virulent viral strain, prolonging virus shedding, and eventually increasing the overall mortality rate of an influenza pandemic (15).

Hence, both diabetes and obesity impair the immune response to viral infections like influenza and influenza vaccination through alterations of the cellular immune system (16). Studies so far suggest that diabetics, as well as subjects with obesity, are at a greater risk of hospitalization and increased complications from influenza (17, 18). Compared with vaccinated healthy-weight adults, vaccinated obese adults have twice the risk of influenza or influenza-like illness despite equal serological response to vaccination (19). This should be considered one of the challenges to be overcome in vaccine development and/or medications to combat this virulent respiratory virus and prevent future epidemics similar to COVID-19.

## Hyperinflammation in COVID-19 Infected Patients

Obesity is also associated with chronic low-grade inflammation, dysbiosis, and increased secretion of inflammatory cytokines, including interleukin 6 (IL-6) (20). Elevated plasma levels of pro-inflammatory cytokines are observed in COVID-19 infected patients; in particular, IL-6 and ferritin release have been identified as predictors of fatality (21). Several studies focusing on previous outbreaks of severe influenza confirmed that the mortality caused by organ injury could be reduced by immunomodulatory agents (22, 23). Currently, a study on the safety and efficacy of Tocilizumab is being conducted (ClinicalTrials.gov Identifier: NCT04317092) (24) to assess its capacity to suppress the virally driven hyperinflammation and acute respiratory syndrome caused by COVID-19.

As mentioned previously, COVID-19-related deaths are due to acute respiratory distress syndrome (ARDS); nonetheless, data indicate that the COVID-19 virus is detectable in stool of infected patients, suggesting systemic manifestations (25). In line with this observation, reports indicate abundant expression of ACE2 in absorptive enterocytes of the GI tract (25) and diarrhea as among the most frequent infection symptoms (26). Inflammation of intestinal mucosa may result in increased intestinal permeability with a consequent cascade of events that cause persistent inflammation, worsening the infection-related symptoms. Immune homeostasis is a dynamic process maintained by a complex interplay between the gut microbiota and host mucosal immune system (27). Dysbiosis, defined as imbalances in gut microbial species, is now a well-recognized factor in the pathogenesis of age-associated frailty (28). It is possible that COVID-19 mortality is increased in older patients with comorbidities associated with intestinal dysbiosis, as this could support systemic chronic inflammation in the host. Nonetheless, only future studies will clarify this aspect.

## Can Nutritionally Derived Products Help To Contain/Alleviate COVID-19-Induced Inflammation?

ACE2-expressing epithelial cells are the primary targets of COVID-19. Similarly to other pulmonary viral infections, following primary exposure, the progeny proliferate in the host cells, which consequently die and release their contents. COVID-19 is now able to infect other cells, including alveolar macrophages (29). Activated or infected immune cells secrete excessive pro-inflammatory cytokines and chemokines, fuelling a vicious circle leading to pulmonary tissue damage. Data accumulating from COVID-19 patients indicate that these patients might have a cytokine storm syndrome, with markedly higher levels of IFN-γ, CCL-2, CCL-3, TNF, and the aforementioned IL-6 (30–32).

For optimal functioning of the immune system, an adequate nutritional status is required. This is well-acknowledged from evidence linking nutritional deficiencies to the functionality of the immune system (33). Poor nutrition leads to poor immune defense, and it is frequently associated with impaired immunity and increased susceptibility to infection. Nevertheless, nutrient inadequacies and deficiencies in our habitual diet are common (34), and immune function may be improved by restoring nutrients to recommended levels, increasing resistance to infection, and hastening recovery once infected (35)_._

Furthermore, studies have revealed inadequate micronutrient levels in patients who were hospitalized in the infectious disease department, including thiamine, selenium, zinc, and vitamin B6 deficiencies (36), which were associated with adverse clinical outcomes. Early detection, prevention, and treatment should aim at decreasing the inflammatory response and avoiding the excessive post-inflammatory immune suppression, defined as compensatory response (37), that is observed in many such patients (38).

Various micronutrients are essential for immunocompetence, particularly vitamins A, C, D, E, B2, B6, and B12, folic acid, iron, selenium, and zinc, as well as macronutrients likely omega 3 fatty acids (39) and bioactive components as polyphenols (40).

**Figure 1:**
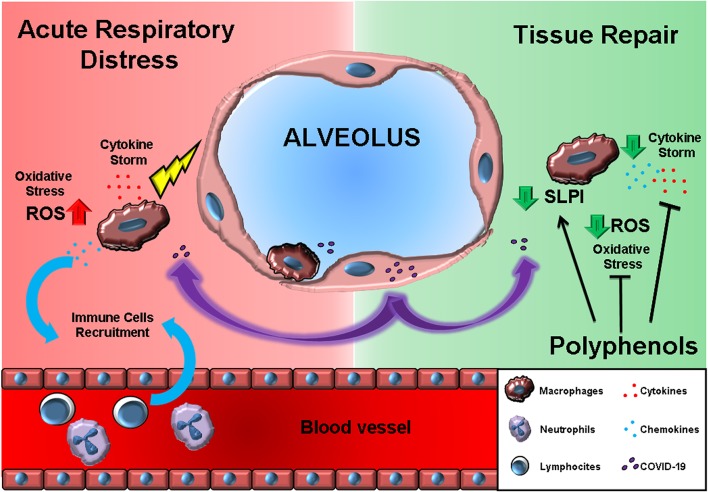
The effects of polyphenols may antagonize the acute respiratory distress induced by macrophage-produced pro-inflammatory cytokine storm. On the left (RED background), COVID-19 infection results in cytokines, and chemokine release by alveolar macrophages. Chemokines further recruit circulating immune cells. On the right (GREEN background), polyphenol exposure can block the immune cell inflammatory program and, at the same time, promote the SLPI release crucial for tissue repair.

During recent years, our groups, together with several others worldwide, demonstrated the ability of nutritionally derived bioactive compounds to suppress inflammatory cytokine release (40–43). In particular, the administration of several plant-derived polyphenols to *in vitro* cultured immune cells suppressed the release of inflammatory cytokines (44). Traditional Chinese herbal medicines for influenza treatment demonstrated potential antiviral activity (45, 46). Polyphenol bioavailability has been long discussed, as many have observed health benefits, but few have observed circulating traces of bioactive compounds (47, 48). Nonetheless, exposure of the epithelial barrier to a polyphenol-rich environment can efficiently activate local immune suppression and tissue repair mechanisms (49), and systemic benefits may be related to the release of circulating microRNA, defined as small non-coding RNA molecules, with anti-inflammatory effects (50). Indeed, even if orally introduced, bioactive dietary factors induce miRNA synthesis, these are packaged into exosomes and released into the bloodstream to act systemically (51). Dietary factors are emerging as anti-inflammatory miRNA promoters able to regulate metabolic functions, inflammation, and oxidation systemically (52–55). Furthermore, herb extracts may combine antiviral, anti-inflammatory, and antioxidant activity, and tissue-repair properties (56, 57) (Figure 1).

**Figure 2:**
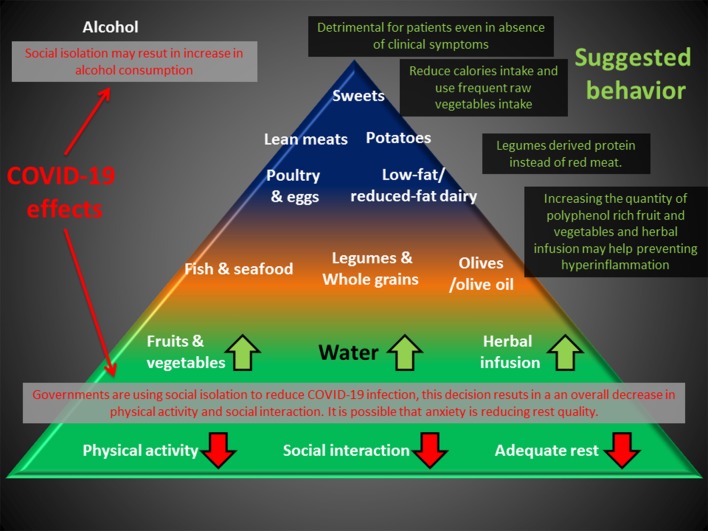
Deleterious effects of the anti-COVID-19 strategy (RED) can impact the base of the Mediterranean diet pyramid. An increase in polyphenol uptake and substitution of meat with legume-derived proteins can help prevent chronic inflammation, and reduction of caloric intake may compensate for the reduction in physical activity. Alcohol consumption is discouraged for healthy people and is detrimental for patients, even in the absence of clinical symptoms.

Altogether, these observations suggest that people may benefit from a correct nutritional intake, particularly during this period of uncertainty. Choosing dietary regimes that may potentially work as adjuvants for preventing undesired hyper-inflammation might be particularly useful for patients with mild signs of infection (Figure 2).

## Discussion

General recommendations for healthy adults over 50 years of age observing a period of lockdown and thus with limited options for physical activity should focus on healthy dietary patterns. These can be generally described as those rich in plant-based foods, including fresh fruits and vegetables, soya, nuts, good sources of antioxidants (58), and omega-3 fatty acids (59) and low in saturated fats and trans fats, animal-derived proteins, and added/refined sugars (60). Moreover, mild energy restriction is recommended for obese and obese-diabetic patients (15). Most of these dietary targets can be met in our country by means of the well-known and traditionally familiar Mediterranean diet (61, 62), which is rich in polyphenols with immune-protective and anti-inflammatory activities, playing an adjuvant role in both prophylaxis and therapy (61). The scientific community is already discussing how to manage future epidemic outbreaks by learning from the current experience (63). Future studies should also focus on the effects of nutrition on immune function, identifying target population subgroups with the most vulnerable immune systems, such as the elderly and those with comorbidities.

## Author Contributions

HC and MC wrote the manuscript.

## Conflict of Interest

The authors declare that the research was conducted in the absence of any commercial or financial relationships that could be construed as a potential conflict of interest.

## Footnotes

^1^Available online at: https://www.worldobesity.org/training-and-events/events/wof-webinar-the-collision-of-two-pandemics-covid-19-and-obesity
